# Axillary schwannoma, preoperative diagnosis on a tru-cut biopsy: Case report and literature review

**DOI:** 10.1016/j.ijscr.2018.09.039

**Published:** 2018-09-29

**Authors:** Hager Aref, Georges A. Abizeid

**Affiliations:** Department of Surgery, International Medical Center, P.O. Box 1716, Jeddah 21441, Saudi Arabia

**Keywords:** CT, computed tomography, MRI, magnetic resonance imaging, ER, emergency room, FNA, fine needle aspiration, CNB, core needle biopsy, Schwannoma, Axillary schwannoma, Preoperative diagnosis, Tru-cut biopsy, Case report

## Abstract

**Introduction:**

Schwannoma is a benign encapsulated tumor of the peripheral nervous system which is mainly located in the head and neck. However, axillary Schwannoma is rare. In this article, the author presents an interesting case of a right axillary Schwannoma, where the diagnosis was preoperatively made on a Tru-cut biopsy.

**Case presentation:**

We report a case of an enlarged right axillary swelling in a sixty-year-old male. Proper radiologic workup was performed. Furthermore, a Tru-cut biopsy was taken which revealed evidence of schwannoma. Having such symptomatic lesion, operative excision was done with the use of a nerve stimulator, which aided in preserving nerve function.

**Conclusion:**

With such non-specific presentation, axillary Schwannoma may be easily missed and mismanaged. A Tru-cut biopsy may provide great help in expert hands, aiding operative planning; likewise in the case we are presenting. Furthermore, utilizing an intraoperative nerve stimulator will facilitate nerve preservation.

## Highlights

• Although Axillary Schwannoma is a rare condition, physicians should recognize it as a part of their differential diagnosis of an axillary swelling.
• Preoperative utilization of Tru-cut biopsy in diagnosing this condition will further help surgeons to plan proper surgical treatment.
• The author recommends utilizing an intraoperative nerve stimulator to facilitate nerve preservation during excision; aiming to provide optimal management and better outcome.

## 1 Introduction

Schwannoma, also known as Neurilemmoma [1], is a benign encapsulated tumor of the peripheral nervous system. It develops from Schwann cell, giving the tumor its name. Schwannoma arises in the nerve sheath. It was first described by Verocay in 1908 [2,3]. This tumor can be found anywhere in the body, but the most common locations are head and neck, accounting for about 25%, with only 5% of the lesions located in the axilla [4]. Schwannoma mostly occurs in the third and fourth decades of life [1,5,6], but there is no sex or racial predilection [7]. It is usually present many years before diagnosis [8]. Secondary to its rarity, many cases can be missed, so it should be kept in mind as one of the differential diagnosis when evaluating an axillary mass [9]. Hence, the diagnosis and management may be challenging to surgeons.

In this article, the author presents an interesting case of a right axillary Schwannoma in a sixty-year-old male, where the diagnosis was preoperatively made on Tru-cut biopsy. This paper has been dictated fulfilling by the SCARE criteria [33].

## 2 Case presentation

**Fig. 1:**
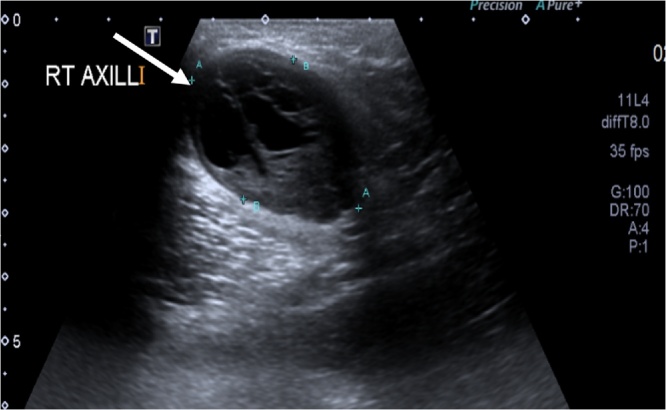
Ultrasound of the right axillary area showing a mass with cystic degeneration.

We present the case of a sixty-year-old male patient, who is a smoker with negative past medical and surgical history. He presented to our surgical clinic, with a right axillary mass which was noted first, three years before presentation. The mass has been slowly increasing in size and becoming painful. There was no history of trauma to the affected area, fever, night sweats, chills or any other systemic symptoms. He only complained of a painful, visible swelling but had no weakness, numbness or loss of function of the right upper limb. He reported no history of any drug intake. Furthermore, family history was unremarkable, he didn’t report any relevant psychosocial history. On **examination**, no skin changes were observed. He had a right axillary mass that is measuring about 4 × 3 cm, which was firm, mobile and tender on palpation. It was nonadherent to the underlying tissue. No palpable left axillary or cervical lymph nodes. Muscle power in all muscles was 5/5; the sensation was intact. Tinel sign was positive with tingling sensation along the shoulder tip. The left axilla was normal. Chest examination was unremarkable as well. Based on the history provided by the patient and the examination findings, our differential diagnosis included axillary lymphadenopathy, lipoma, fibroma, vascular tumors, and paraganglioma. Laboratory investigations were normal. Furthermore, **Ultrasound**-Soft tissue of the right axilla, revealed a subcutaneous, well defined, hypodense lesion, measuring 3.7 × 2.4 cm with evidence of cystic degeneration (Fig. 1). Also, an Ultrasound guided **tru-cut needle biopsy** was performed under complete aseptic technique, with no immediate complications. The **histopathology** sections show a tumor formed of benign-looking spindle cells with Hypercellular and hypocellular areas and vascular hyalinization. Immuno-histo-chemistry of the tumor cells was positive for S100. The diagnosis was right axillary Schwannoma.

**Fig. 2:**
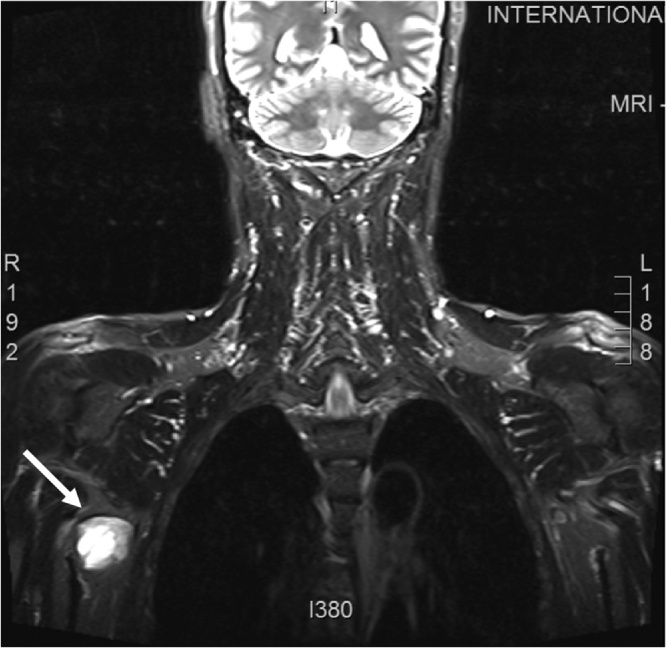
MRI of the right axillary area involved, showing a lesion in the right axilla measuring 3.0 × 3.8 × 2.3 cm, with primary cystic component and irregular thickened wall.

**MRI of the right brachial plexus** revealed an expanding lesion within the right axilla measuring 3.0 × 3.8 × 2.3 cm in maximum dimension, with primary cystic component and an irregular thickened wall that showed significant enhancement after intravenous contrast administration. The lesion is located beneath the axillary vessel. No evidence of osseous infiltration, (Fig. 2). The **whole spine MRI** was also performed to rule out other synchronous lesions. It showed, straightening of the cervical spine, with a diffuse central disc bulge in C3-4, C5-6, C6-7, there were no masses visualized.

He also underwent **neurophysiology testing,** which was normal with no evidence of neuropathy, radiculopathy, or plexopathy of the right upper extremity.

**Fig. 3:**
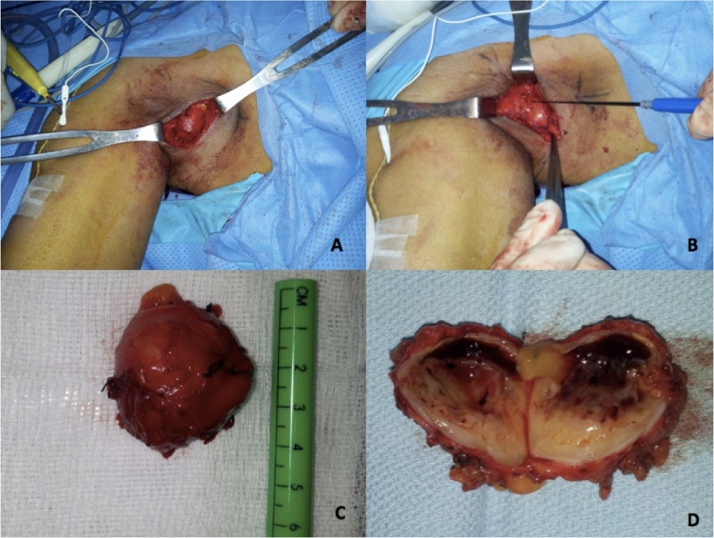
Intraoperative Imaging, A: Axillary incision with subsequent dissection to access the tumor. B: Using the nerve stimulator while operating for careful dissection and avoiding nerve injury, C: The tumor after excision, measuring 4 × 4 cm. D: Gross pathology of the excised tumor, split in half, showing cystic component.

He underwent Exploration of brachial plexus and excision of the tumor. It was performed by the co-authors of this paper. The surgery was performed starting with a transverse incision along the skin line. Dissection carried out through planes, between the borders of Pectoralis Major anteriorly and Latismus Dorsi muscles posteriorly. The tumor was identified, measuring 4 × 4 cm. It was adherent to the musculocutaneous nerve. Using a nerve stimulator, we safely dissected the tumor preserving the nerve nearby. The lesion was completely excised and was sent for histopathology, (Fig. 3).

**Fig. 4:**
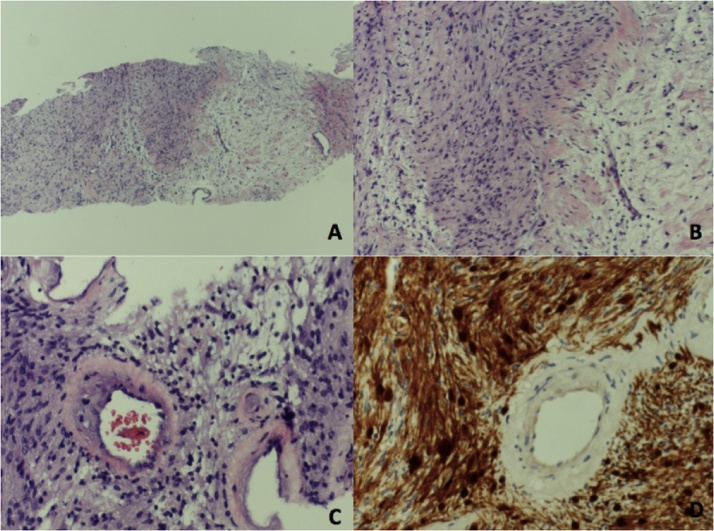
Photomicrograph A: Tumor with hypercellular and hypocellular areas, B: Tumor formed of benign-looking spindle cells, C: Vascular hyalinization, D: Positive S100 immuno-stain.

**Histopathology** confirmed the diagnosis of right axillary schwannoma, with no evidence of malignancy, (Fig. 4).

In the post-operative period, the patient had an uneventful recovery. He was followed up in the clinic and displayed no neurological deficits, his wound has healed, the histopathology findings were discussed and he was satisfied with the care provided.

## 3 Discussion

Schwannoma is a benign, slow-growing tumor of the nerve sheath. It is derived from Schwann cells of the peripheral nerves. It was initially described by Verocay in 1908 [2,3]. Schwannoma constitutes 8% of all soft tissue masses [10,11]. It is considered the most common peripheral nerve tumor [[12], [13], [14]]. Schwannoma affects mainly the head, neck, and extremities. Upper extremity Schwannoma accounts for 12 to 19% and lower extremity schwannoma accounts for 13.5 to 17.5% of all cases [10]. Axillary schwannomas are extremely uncommon [15]. In a study by Gosk et al., studied the surgical treatment of extremities’ schwannomas, they included cases from the year 1985 to 2013, and have reported only 6 cases of axillary schwannoma [16]. Patients with schwannoma usually present in the third to fifth decades of life, and there are no racial or gender differences [17,18]. Surprisingly, the presented case is even older than that; he presented in his sixth decade of life. These tumors are well encapsulated and characterized by their slow, noninfiltrating growth pattern [11,19]. Being solitary and small in size, ranging from 1.5 to 3 cm in diameter, could contribute to the delay in presentation [20,21].

Hence, the patient can present with a noticeable mass, compressive neuropathy or both. Neurologic symptoms usually occur late. Symptoms may be nonspecific, and it may take several years before it gets diagnosed [22].

Similarly, our patient had the axillary mass for three years, which was slowly growing and eventually causing pain, made him seek medical advice. The fact that it is a slow-growing tumor will lead to gradual displacement of nerve fascicles [11,19], eventually resulting in symptoms of nerve compression [23,19]. Fortunately, in the presented case there were no signs of nerve compression caused by the tumor.

Diagnosis and classification of this tumor are challenging. Fine needle aspiration (FNA) is one of the methods of diagnosing schwannoma, although it is difficult to recognize tissue architectural pattern on cytology, it was reported that FNA was used for the diagnosis of schwannoma [24]. They further confirmed the diagnosis by histopathology following surgical excision. A tru-cut biopsy can also be used for the diagnosis of schwannoma. In a case report by Nasrollah et al., core needle biopsy (CNB) was used to diagnose neck schwannoma. It revealed a proliferation of neuronal type consistent with Schwannoma [25]. Hong et al. reported a case of gastric schwannoma where the lesion was diagnosed using tru-cut biopsy [26]. Furthermore, CNB of a suspected peripheral nerve sheath tumor may be performed safely before surgery to confirm the diagnosis [27].

Comparing FNA and CNB in the diagnosis of extremity soft tissue tumors, showed that FNA had 79.17% sensitivity, and 72.7% specificity, while CNB had 81.8% sensitivity, and specificity of about 79.2%. Regarding the accuracy in identifying the correct diagnosis, FNA had a 33.3% accuracy and CNB had a 45.6% accuracy [28].

A retrospective study conducted in 2013, evaluated the sensitivity and specificity of CNB in determining musculoskeletal tumors. Among these tumors, Eleven had schwannomas (Seven benign and four malignant), yet there was no specification of the location. The sensitivity and specificity of CNB were reported to be 95%, and 97%, respectively [29]. CNB was of great use in such cases and has enabled physicians to diagnose schwannoma in situations where radiological imaging and FNA didn’t [30].

Upon searching the literature, to our knowledge, there are no reported cases of axillary schwannoma which is diagnosed, preoperatively by a CNB. In the case described, an Ultrasound guided true-cut needle biopsy was performed with no complications. The histopathology features and Immuno-histo-chemistry of the tumor cells confirmed the diagnosis of an Axillary Schwannoma.

The differential diagnosis included axillary lymphadenopathy, lipoma, fibroma, vascular tumors, and paraganglioma. Schwannoma should also be kept in the differential diagnosis when dealing with an axillary mass.

Treatment of schwannoma depends on the location, size of the lesion, nature: benign or malignant, age and condition of the patient. The gold standard is surgical excision. On the other hand, malignant schwannomas may require radiation therapy and chemotherapy. Fortunately, surgical excision usually results in complete and immediate resolution of symptoms [22,31,32].

Because they are rare and have complex locations, they have diagnostic and therapeutic challenges. On the other hand, axillary Schwannoma was not well described in the literature where only a few similar cases have been reported. Schwannoma is rarely identified pre-operatively based on a radiologic biopsy. In the presented case, the diagnosis of axillary schwannoma was made before the operation, based on Ultrasound guided tru-cut biopsy and the tumor was surgically excised with no residual neurological deficit.

## 4 Conclusion

Axillary Schwannoma is a rare and challenging condition. Its complex anatomical locations could present diagnostic misinterpretation. Because of the non-specific symptoms, many cases can simply be missed and mismanaged. Schwannoma must be kept in mind, and the operation must be planned according to this possibility. A tru-cut biopsy may provide great help in expert hands, aiding operative planning; Likewise, in the case, we are presenting. Furthermore, utilizing an intraoperative nerve stimulator will facilitate nerve preservation.

## Conflict of interest

There is no conflict of interest. The authors did not receive any financial support.

## Funding source

No funds were provided prior to submission.

## Ethical approval

We have received an approval from the ethical committee in our institute’s research centre (International Medical Centre).

## Consent

Written informed consent was obtained from the patient at the clinic. The patient was informed that his case will be written for publication as a case report with the accompanying images. A copy of the written consent is available for review by the Editor-in-Chief of this journal on request.

## Author contribution

-Hager Aref: Literature reviewer; she wrote the article’s manuscript and submitted the paper for publication.

-Georges A. Abizeid: Operating surgeon; he wrote the operative details and part of the discussion, reviewed and edited the manuscript.

## Registration of research studies

researchregistry4412.

## Guarantor

Dr. Georges Abizeid.

## Availability of data and materials

Data were obtained from our institute’s Health Information System, a computer-based in-house system.

## Provenance and peer review

Not commissioned, externally peer-reviewed.
